# Increasing Recombinant Strains Emerged in Norovirus Outbreaks in Jiangsu, China: 2015–2018

**DOI:** 10.1038/s41598-019-56544-2

**Published:** 2019-12-27

**Authors:** Jianguang Fu, Changjun Bao, Xiang Huo, Jianli Hu, Chao Shi, Qin Lin, Jun Zhang, Jing Ai, Zheng Xing

**Affiliations:** 1 Medical School and the Jiangsu Provincial Key Laboratory of Medicine, Nanjing University, Nanjing, China; 2 Key Laboratory of Enteric Pathogenic Microbiology, Ministry of Health, Jiangsu Provincial Center for Disease Control and Prevention, Nanjing, China; 3 Wuxi Center for Disease Control and Prevention, Wuxi, China; 4 Changzhou Center for Disease Control and Prevention, Changzhou, China; 5 Yangzhou Center for Disease Control and Prevention, Yangzhou, China; 6 College of Veterinary Medicine, Department of Veterinary Biomedical Sciences, University of Minnesota at Twin Cities, Saint Paul, Minnesota 55108 USA

**Keywords:** Preventive medicine, Viral transmission

## Abstract

From January 2015 to December 2018, 213 norovirus outbreaks with 3,951 patients were reported in Jiangsu, China. Based on viral RdRp and VP1 genes, eight genotypes, GII.2[P16] (144, 67.6%), GII.3[P12] (21, 9.9%), GII.6[P7] (5, 2.3%), GII.14[P7] (4, 1.9%), GII.4 Sydney[P31] (3, 1.4%), GII.1[P33] (1, 0.5%), GII.2[P2] (3, 1.4%), and GII.17[P17] (16, 7.5%) were identified throughout the study period. These genotypes were further regrouped as GII.R (Recombinant) and GII.Non-R (Non-recombinant) strains. In this report we showed that GII.R strains were responsible for at least 178 (83.6%) of 213 norovirus-positive outbreaks with a peak in 2017 and 2018. Most norovirus outbreaks occurred in primary schools and 94 of 109 (86.2%) outbreaks in primary schools were caused by GII.R, while GII.Non-R and GII.NT (not typed) strains accounted for 6 (5.5%) and 9 (8.3%) norovirus outbreaks, respectively. The SimPlot analysis showed recombination breakpoints near the ORF1/2 junction for all six recombinant strains. The recombination breakpoints were detected at positions varying from nucleotides 5009 to 5111, localized in the ORF1 region for four strains (GII.2[P16], GII.3[P12], GII.6[P7], and GII.14[P7]) and in the ORF2 region for the other (GII.4 Sydney[P31] and GII.1[P33]). We identified four clusters, Cluster I through IV, in the GII.P7 RdRp gene by phylogenetic analysis and the GII.14[P7] variants reported here belonged to Cluster IV in the RdRp tree. The HBGA binding site of all known GII.14 strains remained conserved with several point mutations found in the predicted conformational epitopes. In conclusion, gastroenteritis outbreaks caused by noroviruses increased rapidly in the last years and these viruses were classified into eight genotypes. Emerging recombinant noroviral strains have become a major concern and challenge to public health.

## Introduction

Norovirus has been recognized as the leading cause of acute nonbacterial gastroenteritis outbreaks worldwide^1^. Human noroviruses are classified into at least five genogroups (GI, GII, GIV, GVIII and GIX) which are further subdivided into 35 genotypes^2,3^. The norovirus genome consists of a 7.5 kb single-stranded and positive-polarity RNA segment encoding three open reading frames (ORFs). ORF1 encodes non-structural proteins including the viral RNA-dependent RNA polymerase (RdRp) and ORF2 and ORF3 encode structural proteins VP1 and VP2, respectively^4^. VP1 is composed of shell (S) and protruding (P) domains and the P domain contains both the antigenic sites as well as histo-blood group antigen (HBGA) binding sites^5,6^.

The epidemiology of norovirus is strongly influenced by norovirus evolution through recombination or accumulation of mutations^7^. Recombination often occurs at the ORF1/ORF2 junction that leads to new combinations of capsid and RdRp types, further increasing genetic diversity^8^. These new recombinant strains might have increased fitness and transmissibility over their parental strains^9^. The same capsid genotype can be associated with different RdRp genotypes, which may offer a temporary selective advantage through altering the efficiency of virus replication^2^. To better understand epidemiologic and genotypic trends of evolving norovirus recombinant strains in the field, we examined and analyzed norovirus outbreak data and strains collected between January 2015 and December 2018 in Jiangsu China. Our analysis showed that recombinant strains increased significantly in norovirus outbreaks between 2015 and 2018 and the GII.2[P16] recombinant strains were responsible for most outbreaks. Recombination appeared to be main force driving norovirus evolution in the field in the recent years.

## Results

### Epidemiological features

**Figure 1:**
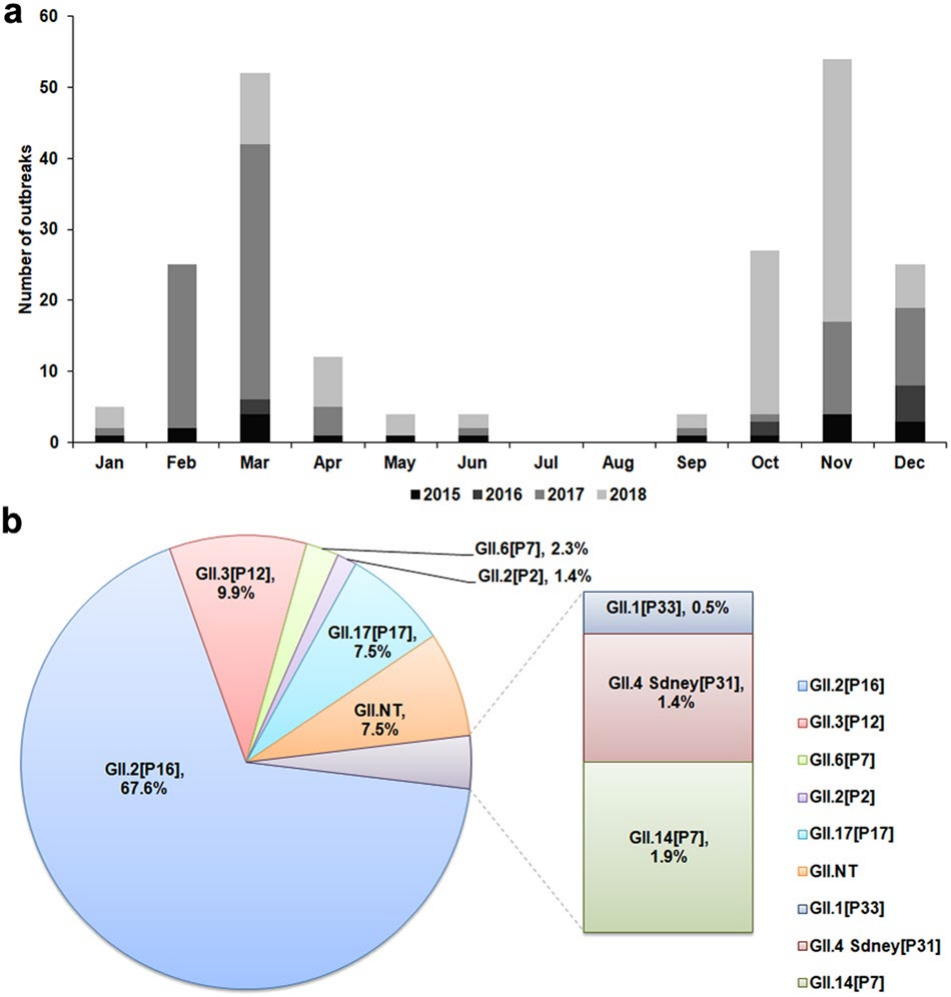
Norovirus outbreaks in Jiangsu, China, 2015–2018. (**a**) Laboratory confirmed number of monthly norovirus outbreaks. (**b**) Distribution of norovirus genotypes detected in norovirus outbreaks.

A total of 213 norovirus outbreaks with 3,951 patients were reported to the Jiangsu CDC from January 2015 to December 2018. Of the 213 outbreaks, 19 (8.9%) occurred in 2015, 9 (4.2%) in 2016, 92 (43.2%) in 2017 and 93 (43.7%) in 2018; 43 (20.8%) were reported in kindergartens, 109 (51.2%) in primary schools, 38 (17.8%) in middle schools, 11 (5.1%) in secondary schools and 11 (5.1%) in other settings; 68 (31.9%) occurred in spring, 5 (2.4%) in summer, 85 (39.9%) in autumn and 55 (25.8%) in winter; 2181 (55.2%) cases were males and 1770 (44.8%) were females. Most outbreaks occurred in the period of season transitions, such as from autumn to winter (November and December) and from winter to spring (February and March). Peaks of culminative outbreaks were observed in March and November whereas no outbreak occurred in July and August, likely due to summer recesses for schools. There were many fewer outbreaks in 2015 and 2016 with the fewest reported in 2016 when cases were reported only in March, October, and December. However, rapid increase of outbreaks in number occurred since February 2017 with most cases reported in that spring. Interestingly in 2018, the cases were fewer in spring and the major peaks of outbreaks occurred in early and late autumn (from October to November). Thus, even though the trend remained similar, the outbreaks in number and peak time differed greatly each year from 2015 through 2018 (Fig. 1a). In addition, there were 7 genogroup I norovirus outbreaks that occurred during this period but were not included in this analysis due to failure to sequencing their RdRp genotypes.

**Figure 2:**
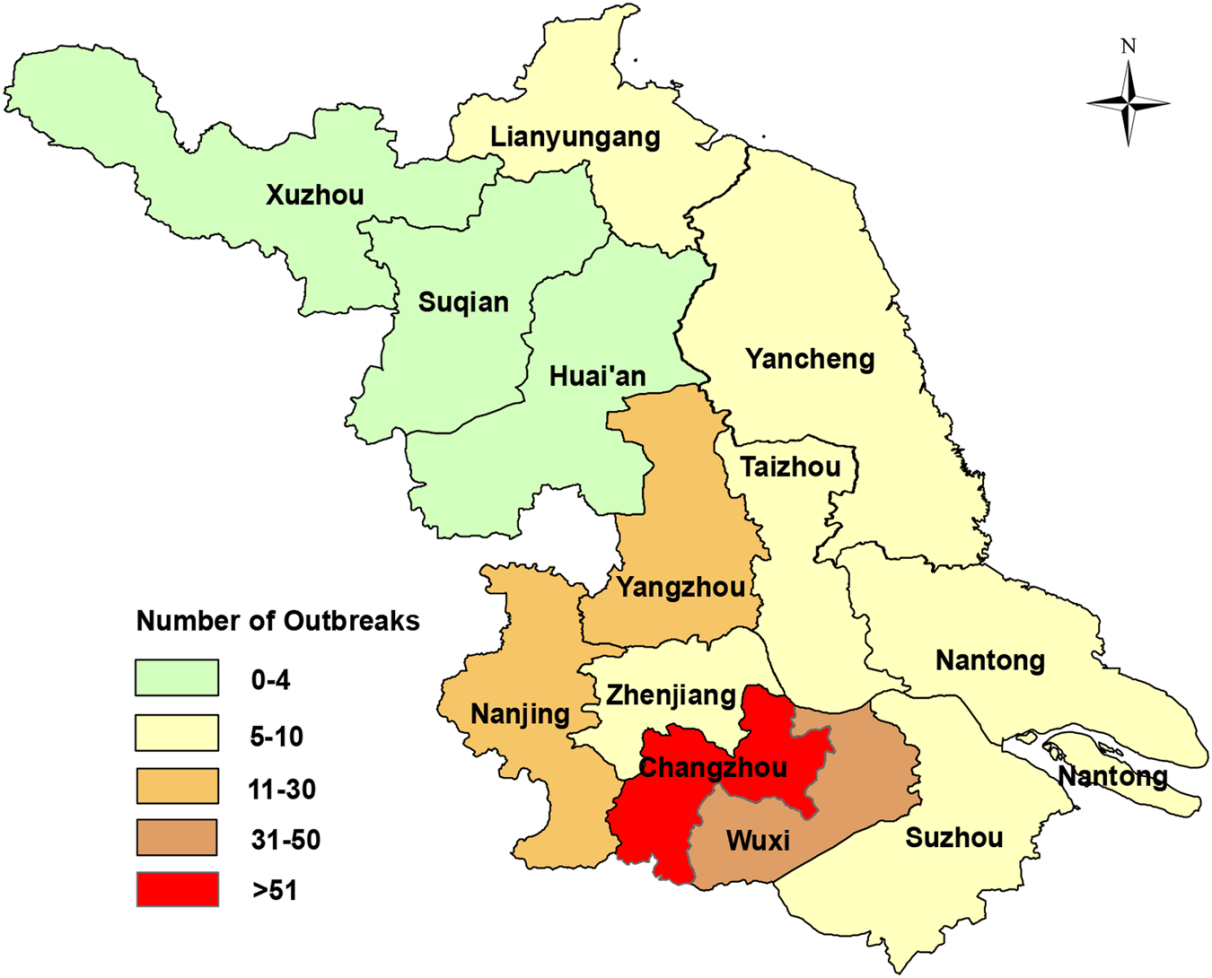
Spatial distribution of norovirus outbreaks in Jiangsu, China, 2015–2018. The outbreaks were shown by geographic locations. The number of the outbreaks was reflected with various colors.

Geographic distribution of the outbreaks is shown in Fig. 2. About 170 (79.8%) outbreaks occurred in four prefecture-level cities in the southwest (Nanjing, Wuxi, Changzhou, and Yangzhou) regions. In contrast, 37 (17.4%) outbreaks were reported in the east regions and only 6 (2.8%) occurred in three cities (Xuzhou, Suqian, and Huai’an) in the northwest regions (Fig. 2).

### Characteristics of the norovirus outbreaks by genotype

Among 213 outbreaks caused by genogroup II norovirus, 197 (92.5%) were genotyped and 16 (7.5%) were not (GII.NT). Based on the RdRp and VP1 sequences, eight genotypes were identified throughout the study period, which included GII.2[P16] (144, 67.6%), GII.3[P12] (21, 9.9%), GII.6[P7] (5, 2.3%), GII.14[P7] (4, 1.9%), GII.4 Sydney[P31] (3, 1.4%), GII.1[P33] (1, 0.5%), GII.2[P2] (3, 1.4%), and GII.17[P17] (16, 7.5%) (Fig. 1b).

To characterize the norovirus outbreaks caused by recombinant strains, eight genotypes were regrouped based on GII.R (Recombinant) or GII.Non-R (Non-recombinant) strains. Six GII.R strains were identified and comprised of GII.2[P16], GII.3[P12], GII.6[P7], GII.14[P7], GII.4 Sydney[P31], and GII.1[P33]. The GII.Non-R strains included the genotypes of GII.2[P2] and GII.17[P17]. Prevalence of GII.R-caused outbreaks increased significantly in recent years from 3.9% (7/178) in 2015 to 48.3% (86/178) in 2017 and GII.R was responsible for at least 178 (83.6%) of 213 norovirus-positive outbreaks with a peak in 2017 and 2018 (Table 1). The number of patients in outbreaks caused by GII.R was 3,181 (80.5%) with a median of 19.5 patients per outbreak.

**Table 1:** Analysis of characteristics associated with GII.Recombinant, GII.Non-Rec and GII.NT outbreaks from 2015 to 2018 in Jiangsu, China.

	GII.Recombinant No. (%)	GII.Non-Rec No. (%)	GII.NT No. (%)	*p*-value
**Years**				
2015	7 (36.9)	10 (52.6)	2 (10.5)	*p* < 0.001
2016	5 (55.5)	2 (22.2)	2 (22.2)	
2017	86 (93.4)	6 (6.6)	0	
2018	80 (86.0)	1 (1.1)	12 (12.9)	
**Number**				
No. of outbreaks	178 (83.6)	19 (8.9)	16 (7.5)	*p* = 0.033
Median no. of cases per outbreak (IQR)	19.5 (10–38.3)	15 (7.5–20.8)	6.5 (5–10)	
**Settings**				
Kindergartens	41 (93.2)	1 (2.3)	2 (4.5)	*p* = 0.011
Primary schools	94 (86.2)	6 (5.5)	9 (8.3)	
Middle schools	30 (78.9)	6 (15.8)	2 (5.3)	
Secondary schools	8 (72.7)	1 (9.1)	2 (18.2)	
Other settings	5 (45.5)	5 (45.5)	1 (9.1)	
**Season**				
Spring, Mar-May	51 (75.0)	12 (17.6)	5 (7.4)	*p* < 0.001
Summer, Jun-Aug	4 (80.0)	1 (20.0)	0	
Autumn, Sep-Nov	77 (90.6)	3 (3.5)	5 (5.9)	
Winter, Dec-Feb	46 (83.6)	3 (5.5)	6 (10.9)	
**Sex**				
Male cases	1743 (79.9)	335 (15.4)	103 (4.7)	*p* = 0.556
Female cases	1438 (81.2)	251 (14.2)	81 (4.6)	

Categorical data are presented as frequencies with percentages; case numbers are presented as the median and interquartile range (IQR); for categorical data, differences among groups were examined using the chi-square test or Fisher’s exact probability test. For continuous data, Kruskal-Wallis Test was used to determine differences among groups.

As shown in Table 1, GII.R strains were the dominant epidemic strains across all settings. Other than the GII.R strains, GII.Non-R and GII.NT strains had similar prevalence rates in kindergartens and primary schools, but GII.Non-R had higher prevalence rates in middle schools than GII.NT. GII.Non-R strains were also the dominant epidemic ones in other settings. Most norovirus outbreaks occurred in primary schools. GII.R strains were responsible for 94 of 109 (86.2%) of norovirus outbreaks in primary schools (Table 1), while GII.Non-R and GII.NT strains were responsible for only 6 (5.5%) and 9 (8.3%) norovirus outbreaks, respectively. Seasonally, GII.R strains were the main genotypes in all seasons with a peak detection rate in autumn, while the peak for GII.Non-R strains were in spring. Of the 3,951 norovirus-positive cases, the number of male cases is higher than that of female cases in each group, although the difference appeared not statistically significant.

### Molecular phylogenetic characteristics of recombinant noroviruses

**Figure 3:**
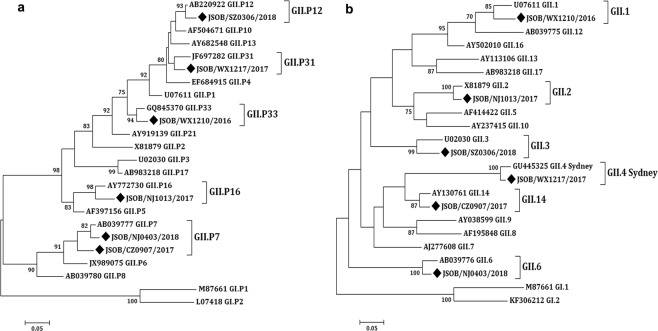
Phylogenetic analyses of the recombinant strain sequences based on partial RdRp and full-length capsid regions (VP1). (**a**) Phylogenetic tree of a 750 bp region of RdRp. (**b**) Phylogenetic tree of complete VP1. The trees were constructed using the Maximum Likelihood analysis and the evolutionary distances were computed using the Kimura 2-parameterþG method available in MEGA 7.0. Bootstrap values (>70%) are shown as percentages derived from 1,000 samplings at the nodes of the tree. The scale bars represent the number of nucleotide substitutions per site. The new norovirus strains reported in this study are indicated with solid black diamonds.

To characterize the potential recombination events of the GII.R strains, a region of 1095 bp in the ORF1/ORF2 junction of the viral genome was amplified by a nested PCR. The sequences were typed by using the calicivirus typing tool (https://norovirus.ng.philab.cdc.gov). The phylogenetic tree was constructed based on partial RdRp gene (750 bp) and capsid gene (365 bp) using the Maximum Likelihood method (Fig. 3a,b). As shown in Fig. 3, six strains had discordant capsid and polymerase genotypes and were considered intergenotype recombinant strains.

**Figure 4:**
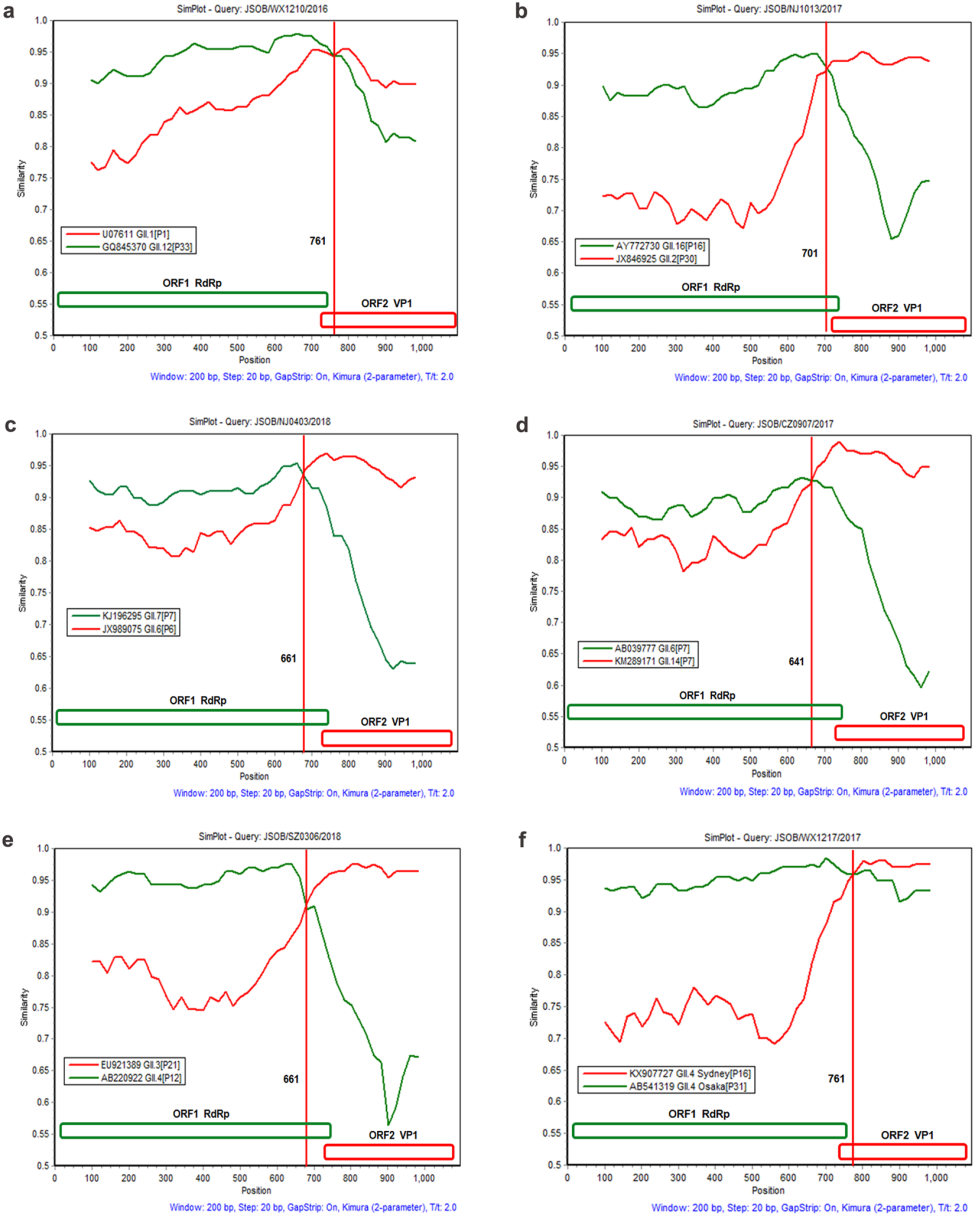
The SimPlot analysis of the recombinant strain sequences. SimPlot was constructed using a Simplot software version 3.5 with a slide window width of 200 bp and a step size of 20 bp. At each position of the window, the query sequence was compared to each of the reference strains. The X-axis indicates the nucleotide positions in the multiple alignments of the NoV sequences; and the Y-axis indicates nucleotide identities (%) between the query sequence and the NoV reference strains.

Since the length of the amplified RdRp fragments from the six recombinant strains was 750 bp long and the corresponding ORF1/2 overlapping regions were 731 to 750 bp, the recombination breakpoints would be near the ORF1/2 junction for all six strains as indicated by the SimPlot analysis (Fig. 4). In fact, the recombination breakpoints were identified at positions varying from nucleotides 641 to 761, corresponding to the nucleotides positioned at 5009 to 5111 in the whole viral genome, localized in the ORF1 region for four strains (GII.2[P16], GII.3[P12], GII.6[P7], and GII.14[P7]) and in the ORF2 for the other two strains (GII.4 Sydney[P31] and GII.1[P33]).

### Phylogeography of GII.14[P7] genotypes

**Figure 5:**
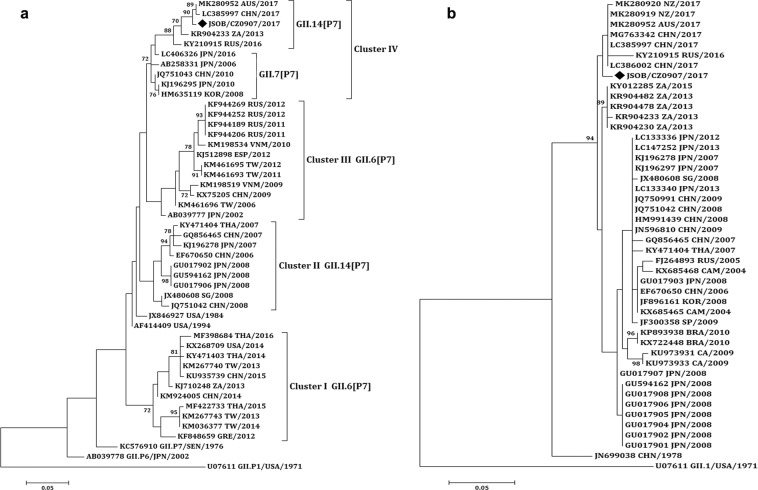
Phylogenetic analyses of the GII.14[P7] sequences based on partial RdRp and VP1. (**a**) Phylogenetic tree of a 313 bp region of RdRp. (**b**) Phylogenetic tree of a 282 bp region of VP1. The trees were constructed using the Maximum Likelihood analysis and the evolutionary distances were computed using the Kimura 2-parameterþG method available in MEGA 7.0. Bootstrap values (>70%) are shown as percentages derived from 1,000 samplings at the nodes of the tree. The scale bars represent the number of nucleotide substitutions per site. The new norovirus strains reported in this study are indicated with solid black diamond.

Of the six recombinant strains, GII.14[P7] was further analyzed because, unlike other strains, it was a rare genotype which did not have an RdRp genotype that belongs to any known RdRp genotypes. According to the phylogenetic analysis, sequences of GII.14[P7] were grouped into four major clusters based on their RdRp genes (Fig. 5).

In detail, as for the RdRp gene, the GII.14[P7] variants identified in the period from 2006 to 2008 fell in Cluster II. The GII.6[P7] variants originating from 2012 to 2016 and from 2002 to 2012 fell in Cluster I and III, respectively. The GII.7[P7] variants and the GII.14[P7] variants from 2013 to 2017 belonged to Cluster IV (Fig. 5a). The GII.14[P7] variant reported here from Jiangsu province was in Cluster IV based on the RdRp trees. As for the VP1 gene, several clusters were observed in the tree, but they did not obtain enough bootstrap support (showed bootstrap support of <70%). The Jiangsu variant was in the same lineage with variants from 2016 to 2017 (Fig. 5b).

Even though the complete VP1 gene of the GII.14[P7] variant in this study has been sequenced, further analysis with VP1 was limited because only a few complete VP1 genes of the GII.14[P7] variants, which were also reported previously within a short period of time, were available in GenBank. On the other hand, three HBGA binding sites of all known GII.14 strains remained conserved, while several amino acid mutations in the predicted conformational epitopes were found^10^ as shown in Table 2. However, a single amino acid change (aa373, D-N) found peripheral to the HBGA-binding site II, which is also located in the predicted conformational epitopes, may have an important effect on the viral antigenicity.

**Table 2:** The sequence analysis of HBGA binding sites and predicted conformational epitopes of GII.14 VP1 protein.

GII.14 strains	HBGA Binding Sites			Predicted Conformational Epitopes		
	Site I (aa347)	Site II (aa374)	Site III (aa439)	aa309	aa365	aa457
**JN699038 CHN/1978**	TRAH	SN**D**F	A**GG**H	LDGSPIDPTDD**V**PAPLGTP	IGQVRFKSSS**N**DFDLHDPTKFTP	EHFYQE**S**APSQS
**AY130761 USA/1999**	TRAH	SD**D**F	A**GG**H	LDGSPIDPTDD**M**PAPLGTP	IGQVRFKSSS**D**DFDLHDPTKFTP	EHFYQE**A**APSQS
**EF547404 JPN/2001**	TRAH	SG**D**F	A**GG**H	LDGSPIDPTDD**M**PAPLGTP	IGQVRFKSSS**G**DFDLHDPTKFTP	EHFYQE**A**APSQS
**EF670650 CHN/2006**	TRAH	SN**D**F	A**GG**H	LDGSPIDPTDD**M**PAPLGTP	IGQVRFKSSS**N**DFDLHDPTKFTP	EHFYQE**A**APSQS
**GQ856465 CHN/2007**	TRAH	SN**D**F	A**GG**H	LDGSPIDPTDD**M**PAPLGTP	IGQVRFKSSS**N**DFDLHDPTKFTP	EHFYQE**A**APSQS
**GU017901 JPN/2008**	TRAH	SN**D**F	A**GG**H	LDGSPIDPTDD**M**PAPLGTP	IGQVRFKSSS**N**DFDLHDPTKFTP	EHFYQE**A**APSQS
**LC133340 JPN/2013**	TRAH	SN**D**F	A**GG**H	LDGSPIDPTDD**M**PAPLGTP	IGQVRFKSSS**N**DFDLHDPTKFTP	EHFYQE**A**APSQS
**JSOB/CZ0907/2017**	TRAH	SN**D**F	A**GG**H	LDGSPIDPTDD**M**PAPLGTP	IGQVRFKSSS**N**DFDLHDPTKFTP	EHFYQE**A**APSQS

The HBGA-binding interfaces are composed of three amino acid motifs that are indicated with sites I, II, and III. The predicted conformational epitopes are also composed of three amino acid components with residues, bold and underlined, that indicate changes. Numbers indicate the starting position of amino acid components, which is located on the norovirus HK74 genome (GenBank accession No. JN699038).

## Discussion

Recombination of human noroviruses is an important mechanism to generate genetic diversity and recombinant strains are frequently detected, particularly between pandemic peaks^11^. From January 2015 to December 2018, norovirus outbreaks caused by recombinant strains increased rapidly in number with a peak in 2017 and 2018. Most outbreaks were attributed to the emergence of GII.2[P16] variants, which were responsible for 67.6% (144/213) of all norovirus outbreaks. During the winter of 2016–2017, the GII.2[P16] strain suddenly emerged and rapidly became the predominant genotype throughout mainland China and Japan^12,13^. During the same period, however, GII.4 Sydney[P16] was the predominant strain in the United States, Australia, and New Zealand^11,14^. GII.P16 polymerases have also been found to recombine with GII.3 and GII.13 capsids, but the P16 polymerase sequence associated with GII.2 capsids is almost identical to the P16 sequences that harbor the GII.4 Sydney capsids^11,14,15^. Emergence of GII.P16 strains indicates that the viral RNA polymerase confirms that ORF1 sequences play a more important role in predominance of certain but not all emerging recombinant genotypes.

To understand how recombination occurred among norovirus, more analyses have been carried out on GII.4 and GII.3 strains for rare occurrence of recombination events in the past. The GII.4 norovirus had been the predominantly detected variant worldwide since 1995. Its capsid protein continuously underwent epochal evolution by emergence of one antigenically distinct GII.4 strain approximately every 3–5 years^8,14^. However, this trend in antigenic evolution did not continue with the GII.4 Sydney[P16] viruses that emerged in 2015. The antigenic domains in the capsid did not evolve with any amino acid changes between the GII.4 Sydney[P16] strains and GII.4 Sydney[P31] strains, although the VP1 sequences of GII.4 Sydney[P16] strains formed a new cluster^11,14,16^. In contrast, GII.3 strains evolved earlier through recombination, which became common genotypes in sporadic infection^17^ and ranked only second to the annual GII.4 epidemic strains in China^18^. In this study, GII.3 was also the second genotype in number causing the outbreaks. Since 2000, most GII.3 noroviruses have become recombinant strains, which possessed a non-GII.3 RdRp genotype. The common types of polymerase recombinants with GII.3 were GII.P12, GII.P16, and GII.P21 (formerly termed GII.Pb)^11,19^. The recombinant strains increased circulation, suggesting that recombination may have contributed to viral immune escape or conferred higher virological fitness^14^ for maintaining the fitted strains or genotypes in human population.

Schools were the main sites for outbreaks, especially in primary schools, which was proportionally higher than the others significantly. There was no outbreak in July and August due to schools’ summer recesses, similar to those previously reported in Shanghai, China, where most outbreaks occurred in kindergartens (48.3%) and primary schools (45.0%). In contrast in Australia, Europe, and the United States, most outbreaks occurred in long-term care facilities, followed by hospitals or restaurants, while outbreaks in schools accounted for only a small fraction. The GII.4 viruses, identified as the most predominant genotypes, were more common in outbreaks in health-care facilities compared to other genotypes^9,14,16^.

Noroviruses are classified into genogroups and genotypes based on amino acid homology in the RdRp and VP1 proteins and some genotypes consist of various subclusters (such as GII.3, GII.4 and GII.6). However, no specific criteria have been applied to classify GII.14[P7] strains within variant types^20,21^. In this study, we subdivided the GII.14[P7] strains into several clusters according to the GII.P7 and GII.14 reference strains. Generally one VP1 genotype of noroviruses combine with one or more polymerase genotypes and the resultant strains usually contain the original polymerase genotypes. For example, the GII.3 VP1 genotype could combine with several RdRp genotypes, such as GII.3[P3], GII.3[P12], GII.3[P16], and GII.3[P21], and the resultant recombinant strains usually possess the original GII.P3 RdRp genotypes. However, the VP1 of GII.14 genotype seems to combine only with GII.P7 RdRp genotype, which results in recombinant strains without possessing the original GII.P14 RdRp genotype. On the contrary, the RdRp of GII.P7 could recombine with other VP1 genotypes, such as GII.6[P7] variants, in addition to GII.14^22^. Although we could find GII.P6 genotypes in RdRp region, no GII.P14 genotypes has ever been found. Our data also show that there were several substitutions in the predicted conformational epitopes compared with the ancestral strain, and one of them was peripheral to the HBGA-binding site II. These results suggest that the recombinant strain was evolving slowly and continuously to achieve long-term fitness and stability in the population.

In summary, this study leverages data from two surveillance systems (EPHEIM and NOSS) to provide a comprehensive analysis of norovirus recombinant strains from both the laboratory and epidemiologic perspectives. The results showed that the proportion of recombinant strains increased significantly in the norovirus outbreaks between 2015 and 2018 in Jiangsu, while the GII.2[P16] recombinant strains were accountable for the majority of the outbreaks. Although antigenic drift and recombination are regarded as the main mechanisms for norovirus evolution, constantly increasing proportion of recombinant strains seems to suggest that viruses are more likely to evolve via recombination recently. On the other hand, we have to bear in mind the possibility that more recombinant strains are detected nowadays probably due to improved detection protocols. The current standard for genotyping includes polymerase and capsid genotypes, for example, while in the past years only the capsid or polymerase region was typed by many laboratories. Retyping the strains collected in the past with the current protocol should be able to deal with this concern unequivocally. Increased surveillance for early identification of potential pandemic variants would provide warning to public health sectors so that they could formulate effective preventive and control measures in time.

## Methods

### Sample collection and ethics statement

Two systems, the Emergent Public Health Event Information Management System (EPHEIM) and the norovirus Outbreak Surveillance System (NOSS), had been used to report noroviruses through outbreak-based surveillance in Jiangsu province. An outbreak was defined as to have at least 20 cases within one week or 5 cases within three days with symptoms including vomiting and/or diarrhea. Patient samples positive for norovirus were submitted to the laboratory of Jiangsu provincial Center for Disease Control and Prevention (CDC) for further analysis. To characterize temporal and spatial distribution of outbreaks, hierarchical mapping was carried out with ArcGIS software (version 10.0; ESRI, Redlands, CA). This study was approved by the Institutional Review Board of Jiangsu CDC with the approval protocol No. JSCDCLWLL2019002. Written informed consent was obtained according to the guidelines of the National Ethics Regulation Committee.

### Norovirus genotyping

Norovirus-positive samples were genotyped in both the ORF1 (RdRp) and ORF2 (capsid VP1) regions. A region of 1,095 bp in the ORF1/ORF2 junction of the viral genome was obtained by RT-PCR using a semi-nested specific primer set as previously described^23^. The genotypes were determined by using the norovirus automated genotyping tool (http://www.rivm.nl/mpf/norovirus/typingtool) and human calicivirus typing tool (https://norovirus.ng.philab.cdc.gov).

The complete VP1 genomic fragments (1.7 kb) of the six recombinant strains were amplified with a semi-nested PCR GII-specific primer set (COG-2F/VN3T20 in the first-round PCR and G2SKF/VN3T20 for the second-round PCR) as previously described^23^. Next, the ORF1/ORF2 junction fragment and the complete VP1 genomic fragment were PCR-ligated through splicing into a 2.4 kb genomic fragment which contained a complete capsid sequence and partial RdRp sequence. All PCR products were purified and subsequently sent to the Sangon Biotech (Shanghai, China) Company for Sanger sequencing.

### Sequences analysis

All nucleotide and amino acid (aa) sequence alignments were performed using Bioedit and MEGA 7.0 software^24^. Phylogenetic trees were constructed using the Maximum Likelihood algorithm with 1,000 bootstrap replicates and a Kimura2-parameter model in MEGA 7.0 with norovirus reference sequences obtained from the GenBank database. Nucleotide sequences obtained from clinical samples were deposited in GenBank under the accession numbers from MK614059 to MK614064.

In order to verify the recombination event, the 2.4 kb genomic fragments, which were constructed by PCR as mentioned earlier and contained the ORF1/ORF2 junction region, were analyzed along with the reference strains obtained from GenBank by using a Simplot software v.3.5.1. The SimPlot analysis was performed by setting the window width and the step size to 200 bp and 20 bp, respectively.

### Statistical analysis

Categorical data were presented as frequencies with percentages. Case numbers were presented as the median and interquartile range (IQR). For categorical data, differences among groups were examined using the chi-square test or Fisher’s exact probability test. For continuous data, Kruskal-Wallis Test was used to determine differences among groups. *p* < 0.05 was considered to indicate a statistically significant difference.

## References

[1] Ahmed SM (2014). Global prevalence of norovirus in cases of gastroenteritis: a systematic review and meta-analysis. Lancet Infect Dis.

[2] de Graaf M, van Beek J, Koopmans MP (2016). Human norovirus transmission and evolution in a changing world. Nat Rev Microbiol.

[3] Chhabra P (2019). Updated classification of norovirus genogroups and genotypes. J Gen Virol.

[4] Vongpunsawad S, Venkataram Prasad BV, Estes MK (2013). Norwalk Virus Minor Capsid Protein VP2 Associates within the VP1 Shell Domain. J Virol.

[5] Shanker S (2011). Structural analysis of histo-blood group antigen binding specificity in a norovirus GII.4 epidemic variant: implications for epochal evolution. J Virol.

[6] Tan M, Jiang X (2010). Norovirus gastroenteritis, carbohydrate receptors, and animal models. PLoS Pathog.

[7] Bull RA, Tanaka MM, White PA (2007). Norovirus recombination. J Gen Virol.

[8] White PA (2014). Evolution of norovirus. Clin Microbiol Infect.

[9] van Beek J (2018). Molecular surveillance of norovirus, 2005-16: an epidemiological analysis of data collected from the NoroNet network. Lancet Infect Dis.

[10] Kobayashi M (2016). Molecular evolution of the capsid gene in human norovirus genogroup II. Sci Rep.

[11] Lindesmith LC (2018). Antigenic Characterization of a Novel Recombinant GII.P16-GII.4 Sydney Norovirus Strain With Minor Sequence Variation Leading to Antibody Escape. J Infect Dis.

[12] Ao Y (2017). Norovirus GII.P16/GII.2-Associated Gastroenteritis, China, 2016. Emerg Infect Dis.

[13] Nagasawa K (2018). Phylogeny and Immunoreactivity of Norovirus GII.P16-GII.2, Japan, Winter 2016-17. Emerg Infect Dis.

[14] Lun JH (2018). Recombinant GII.P16/GII.4 Sydney 2012 Was the Dominant Norovirus Identified in Australia and New Zealand in 2017. Viruses.

[15] Tohma K, Lepore CJ, Ford-Siltz LA, Parra GI (2017). Phylogenetic Analyses Suggest that Factors Other Than the Capsid Protein Play a Role in the Epidemic Potential of GII.2 Norovirus. mSphere.

[16] Burke RM (2018). The Norovirus Epidemiologic Triad: Predictors of Severe Outcomes in US Norovirus Outbreaks, 2009-2016. J Infect Dis.

[17] Mahar JE, Bok K, Green KY, Kirkwood CD (2013). The importance of intergenic recombination in norovirus GII.3 evolution. J Virol.

[18] Jin M (2008). Emergence of the GII4/2006b variant and recombinant noroviruses in China. J Med Virol.

[19] Xue L (2017). Comparative phylogenetic analyses of recombinant noroviruses based on different protein-encoding regions show the recombination-associated evolution pattern. Sci Rep.

[20] Chan-It W, Thongprachum A, Okitsu S, Mizuguchi M, Ushijima H (2014). Genetic analysis and homology modeling of capsid protein of norovirus GII.14. J Med Virol.

[21] Vinje J (2015). Advances in laboratory methods for detection and typing of norovirus. J Clin Microbiol.

[22] Dong X (2019). Should we pay attention to recombinant norovirus strain GII.P7/GII.6?. J Infect Public Health.

[23] Fu J (2015). Emergence of a new GII.17 norovirus variant in patients with acute gastroenteritis in Jiangsu, China, September 2014 to March 2015. Euro Surveill.

[24] Kumar S, Stecher G, Tamura K (2016). MEGA7: Molecular Evolutionary Genetics Analysis Version 7.0 for Bigger Datasets. Mol Biol Evol.

